# Maternal Amino Acid Mixtures Supplementation during Late Gestation and Lactation Improved Growth Performance of Piglets through Improving Colostrum Composition and Antioxidant Capacity

**DOI:** 10.3390/antiox11112144

**Published:** 2022-10-29

**Authors:** Xiongkun Yuan, Xiangyu Zhang, Yujun Wu, Dongsheng Che, Hao Ye, Yu Pi, Shiyu Tao, Junjun Wang, Dandan Han

**Affiliations:** 1 State Key Laboratory of Animal Nutrition, College of Animal Science and Technology, China Agricultural University, Beijing 100193, China; 2 College of Animal Science and Technology, Jilin Agricultural University, Jilin 130118, China; 3 College of Animal Science and Technology, Huazhong Agricultural University, Wuhan 430070, China

**Keywords:** sow, piglet, amino acid mixture, antioxidant capacity, growth performance

## Abstract

During late gestation and lactation, oxidative stress in sows can affect their health and reproductive performance. Supplemental amino acid contributes to the antioxidant capacity of pigs. This study was conducted to evaluate the effects of different combinations of Gln, Leu and γ-GABA (amino acid mixtures, AAMs) during late gestation and lactation on the performance of the sows and their offspring. Fifty large white × landrace sows were randomly assigned to 5 groups (n = 10), including a control group and four AAMs groups (AAMs1, Gln + Leu; AAMs2 (Gln + GABA; AAMs3, Leu + GABA; AAMs4, Gln + Leu + GABA). AAMs supplementation improved the antioxidant capacity of sows, including significantly enhanced total antioxidant capacity in AAMs2, 3 and 4 groups and reduced malonaldehyde concentration in AAMs1, 3 and 4 groups. Additionally, all AAMs significantly increased lactoprotein, total solid and IgA levels of colostrum in sows during lactation. Average body weight of piglets on day 21 after birth in all AAMs groups were significantly increased. Furthermore, the significantly increased total antioxidant capacity was observed in the piglets of every AAMs group. In conclusion, supplementing AAMs during late gestation and lactation improved the antioxidant capacity of sows and colostrum composition, thereby enhancing antioxidant status and the growth performance of piglets. This study provides the possibility of maternal amino acid mixtures to improve the productivity of the swine industry.

## 1. Introduction

Sows undergo placenta formation, rapid fetal development, parturition and lactation during the reproductive cycle. During late gestation, the rapid growth and development of the fetus results in the mother maintaining a high catabolic state [1]. In addition to their diet, sows break down their own fat for their fetuses [2]. After delivery, sows maintain a catabolic state due to the need for synthetic milk [3]. Under normal conditions, cell oxidative metabolism will generate a small amount of reactive oxygen species (ROS). However, during late gestation and lactation, the greatly increased metabolic intensity of sows can induce oxidative stress [4]. This process is characterized by a large accumulation of ROS in the body, including large amounts of superoxide and hydrogen peroxide produced by the placenta and mammary gland [5]. Oxidative stress occurs when the amount of ROS produced in the body exceeds the neutralizing capacity of antioxidant enzymes such as glutathione peroxidase (GSH-PX) and superoxide dismutase (SOD) [6,7]. Excessive ROS can cause oxidative damage to proteins, lipids and DNA, ultimately disrupting cellular function. It has been shown that sows have elevated levels of oxidative stress markers ROS, thiobarbituric acid reactants and 8-hydroxy-2 deoxyguanosine in serum during late gestation and early lactation [2]. Oxidative stress in sows not only reduces reproductive performance but also leads to reduced feed intake, prolonged negative energy balance, deterioration of body status and reduced milk production during lactation [8]. Therefore, it is important to develop effective nutritional interventions for oxidative stress in sows.

Amino acids (AA), as the basic material of protein for animal nutrition, are actively involved in a series of physiological processes such as protein metabolism, nitrogen balance and enzyme synthesis [9,10]. Many studies have shown that amino acids have an antioxidant function and can relieve the oxidative stress of the body. Glutamine (Gln) is a rate-limiting precursor for the synthesis of glutathione, an important antioxidant in the body [11]. Maternal Gln supplementation could improve the intestinal development of piglets by inhibiting the expression of miR-29a and promoting the expression of the tight junction protein [12,13]. Supplementation with Gln could increase the content of SOD and decrease the content of malonaldehyde (MDA) in the serum of weaned piglets [14]. Leucine (Leu) is an essential and directly linked amino acid (BCAA) whose physiological functions include regulating protein metabolism, providing oxidative energy and enhancing antioxidant enzyme activity [15,16]. Maternal Leu supplementation could promote skeletal muscle protein synthesis and alleviate intrauterine growth restriction in fetuses through mTOR/S6 K1 pathways [17,18]. A study has shown that Leu supplementation can increase the expression of antioxidant genes in the liver of finishing pigs under heat stress, which may be related to the Keap1-Nrf2 signaling pathway [19]. Gamma-aminobutyric (γ-GABA), as a natural non-protein amino acid, can significantly increase the content of GSH-PX in serum and reduce the levels of MDA and adrenocortical hormone, thereby alleviating transport stress in growing–finishing pigs [20]. Therefore, amino acids have potential application value in reducing oxidative stress and improving productive performance in sows.

Diets supplemented with Gln, Leu or γ-GABA, individually, were reported to be effective in improving the performance of pigs [12,13,14,15,16,17,18,19,20]. However, it remains to be explored if different combinations of these three amino acids are effective or not for reducing oxidative stress in sows and enhancing reproductive performances. The present study aimed to investigate the effects of maternal dietary supplemented with Gln, Leu and γ-GABA combinations from late gestation to the end of lactation on performance and the antioxidant and immunological status of sows and their piglets.

## 2. Materials and Methods

### 2.1. Reagents

L-glutamine and L-leucine were purchased from Henan Mengzhide Trading Co., Ltd., Zhengzhou, China. γ-aminobutyric acid was purchased from Suzhou Lejuan Biotechnology Co., Ltd., Suzhou, China. L-alanine (Ala) was purchased from Hebei Huayang Biotechnology Co., Ltd., Hengshui, China.

### 2.2. Animals and Treatments

Fifty large white × landrace sows (average BW, 182.94 ± 3.04 kg; parity, 1.11 ± 0.38) on day 85 of gestation were divided into five groups, with ten sows in each group, according to the principle of similar BW and parity. The basal diet was formulated on the basis of the nutritional needs of pregnant and lactating sows according to the NRC 2012 (Table 1). Based on the basal diet, the control group was supplemented with 1.05% Ala, and the other four groups were supplemented with AAMs1 (basal diet + 0.55% Gln + 0.44% Leu + 0.01% Ala), AAMs2 (basal diet +0.55% Gln + 0.01% GABA + 0.29% Ala), AAMs3 (basal diet + 0.44% Leu + 0.01% GABA + 0.67% Ala) and AAMs4 (basal diet + 0.55% Gln + 0.44% Leu + 0.01% GABA), respectively. Treatments were started from day 85 of gestation to day 21 of lactation. Sows were housed individually in pregnant house from day 85 to 109 of gestation and transferred to the farrowing house individually on day 110 of gestation until weaning on day 21 of lactation. Each sow was kept in separate pens. The temperature of the farrowing room was strictly controlled at 20 °C, and the room light cycle was set at 06:00 to 16:00. Sows during late gestation were restricted to a 3.5 kg daily diet. The sows had ad libitum access to feed and water during the entire lactation period. The total daily ration and feed intake for each sow were recorded separately. Piglets were provided free access to milk and treated according to routine management. Creep feed was supplied to piglets on day 8 postpartum. All sows were delivered within two weeks. The neonatal piglets were weighed within 12 h after birth. Litters were standardized to achieve an approximately similar number of suckling piglets per litter by cross-fostering within each group.

### 2.3. Litter Performance and the Feed Intake of Sows

Litter size at birth, birth weight and litter birth weight were used to evaluate the litter performance of sows. Feed intake during lactation was recorded.

### 2.4. Growth Performances of Neonatal Piglets

Average litter weight and average body weight on days 7, 14 and 21 after birth were recorded and calculated for the ADG and average daily litter gain (ADLG).

### 2.5. Sample Collection

Blood samples were collected from sows and piglets on day 110 of gestation and days 7, 14 and 21 of lactation, respectively. Six piglets were sampled from different litters in each treatment group according to the average body weight. The serum was separated by centrifugation at 3000 × rpm for 15 min and stored in 1.5 mL aliquots at −80 °C. Within 5 min of parturition, a total of 25 mL of colostrum was extruded from the front, middle and rear teats of sows and stored at −20 °C.

### 2.6. Determination of Amino Acid

The concentrations of free amino acids in serum and colostrum were measured by ultra-high-performance liquid chromatography. Samples were centrifuged at 5000 × rpm for 5 min at 4 °C. 200 μL supernatant was transferred to 1.5 mL centrifuge tube. Thereafter, 8 μL of phenylpropanoid solution and 800 μL of methanol were added in sequence and kept at 4 °C for 2 h. The solution was centrifuged at 14,000 rpm for 10 min at 4 °C, and then 200 μL of the supernatant was collected and dried at 55 °C for 3 h. A total of 100 μL boric acid was added to dissolve the dried sample. A total of 10 μL of dissolved sample was mixed with 50 μL boric acid buffer and 20 μL derivatizing reagent. After one minute, the tube was sealed and heated at 55 °C for 10 min, and the content of free amino acids was determined by ultra-high-performance liquid chromatography (Agilent1200, Agilent, Santa Clara, CA, USA).

### 2.7. Assessment of Colostrum Composition

Milk fat, lactoprotein, lactose and total solids were assessed by a Milk Component Somatic Cell Count Tester (YQ1-55, Bentley Instruments, Chaska, MN, USA). Immunoglobulin A (IgA), Immunoglobulin G (IgG) and Immunoglobulin M (IgM) of colostrum were tested by ELISA assay kits (Prodia Diagnostics Company, Bötzingen, Germany). Total antioxidant capacity (T-AOC), total superoxide dismutase (T-SOD) and GSH-PX and MDA levels in serum were analyzed by assay kits (Nanjing Jiancheng Biotechnology Company, Nanjing, China). T-AOC was assessed by the ABTS method. T-SOD was assessed by the hydroxylamine method. GSH-PX was assessed by an assay kit with the colorimetric method. The MDA concentration was assessed by an assay kit with the thiobarbituric acid method. Before analyzing antioxidant indicators, we removed the cellular component of the colostrum by cryogenic centrifugation at 3000 × rpm for 15 min.

### 2.8. Determination of Serum Biochemical Parameters

Alanine aminotransferase (ALT), aspartate aminotransferase (AST) and alkaline phosphatase (ALP) were determined by using an Automatic Biochemical Detector (Hitachi 7600, Hitachi, Tokyo, Japan). IgG and IgM were tested by using assay kit from Prodia Diagnostics Company, German. Growth hormone (GH) and insulin-like growth factor 1 (IGF-1) were measured by using ELISA test kits (Beijing Konka Hongyuan Biotechnology Co., Ltd., Beijing, China). D-lactate (D-LA) in serum were tested by using assay kits from Nanjing Jiancheng Biotechnology Company, China. T-AOC, T-SOD, GSH-PX and MDA levels in serum were analyzed by assay kits (Nanjing Jiancheng Biotechnology Company, Nanjing, China).

### 2.9. Statistical Analysis

Data were analyzed statistically using SPSS 21.0 (Chicago, IL, USA). Individual sows and total piglets in a litter were respectively regarded as an experimental unit to analyze the litter performances of sows and the growth performance of piglets. Six piglets from different pens per group close to the group average body weight were considered as the unit of analyses for the difference in serum indicators. One-way ANOVA and Duncan test were used to analyze the significance of differences between groups for data satisfying a normal distribution, and a nonparametric test was used to analyze the significance of differences between groups for data not satisfying a normal distribution. Results are expressed as means ± SEM. Probability values less than 0.05 indicate a significant difference, and probability values between 0.05 and 0.1 indicate a trend.

## 3. Results

### 3.1. Effects of AAMs Supplementation on Litter Performance and the Feed Intake of Sows

We first investigated the effects of AAMs supplementation on the litter performance and feed intake of sows. As shown in Table 2, there was no significant difference in the number of piglets born, born alive, healthy piglets, birth weight and birth litter weight. However, sows from the AAMs4 group had an higher average daily feed intake during lactation (*p* < 0.05).

### 3.2. Effects of AAMs Supplementation on Serum Free Amino Acid Concentrations of Sows

The results of amino acid concentration in the serum of sows on day 110 of gestation are shown in Table 3. Maternal Gln and Leu supplementation increased the serum concentrations of Leu and lysine (Lys) (*p* < 0.05). The concentration of Lys was higher in the AAMs2 group (Gln + γ-GABA) than that of the control group (*p* < 0.05). Leu and γ-GABA supplementation enhanced the concentrations of Leu, glutamic acid (Glu), arginine (Arg) and Lys compared to the control group (*p* < 0.05). The AAMs4 group (Gln + Leu + γ-GABA) had higher concentrations of Leu, Glu, phenylalanine (Phe) and Lys than the control group (*p* < 0.05).

### 3.3. Effects of AAMs Supplementation on the Antioxidant Status of Sows

The results of antioxidant status in the serum of sows on day 110 of gestation are presented in Table 4. Compared with the control group, dietary AAMs supplementation improved the antioxidant status of sows, as indicated by the increased levels of T-AOC and GSH-PX in the AAMs2 group (Gln + γ-GABA), as well as reduced MDA content and enhanced T-AOC and T-SOD levels in the AAMs3 group (Leu + γ-GABA). In addition, sows from the AAMs4 (Gln + Leu + γ-GABA) group had higher levels of T-AOC and GSH-PX and lower concentration of MDA than the control group (*p* < 0.05).

### 3.4. Effects of AAMs Supplementation on Colostrum Composition

**Figure 1:**
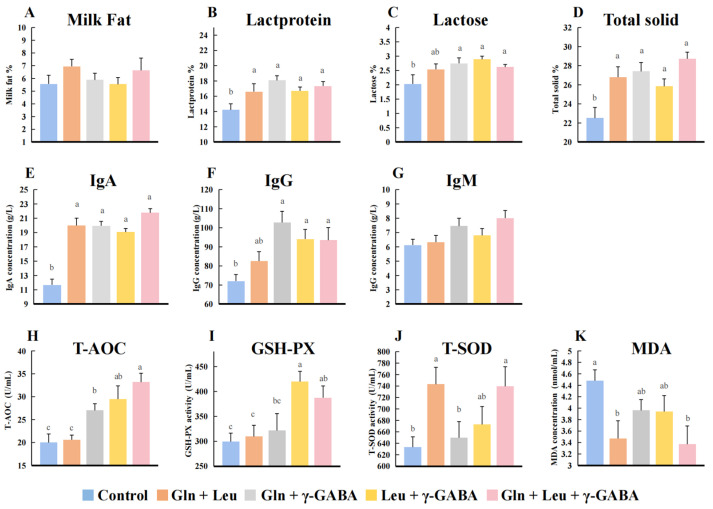
Effects of maternal AAMs supplementation during late gestation and lactation on the composition and antioxidant indices of colostrum (n = 10). (**A**), milk fat; (**B**), lactoprotein; (**C**), lactose; (**D**), total solid; (**E**), IgA; (**F**), IgG; (**G**), IgM; (**H**), T-AOC; (**I**), GSH-PX; (**J**), T-SOD; (**K**), MDA. IgA, immunoglobulin A; IgG, immunoglobulin G; IgM, immunoglobulin M; T-AOC, total antioxidant capacity; GSH-PX, glutathione peroxidase; T-SOD, total superoxide dismutase; MDA, malondialdehyde. All data are presented as mean ± SEM. ^a–c^ The values with different letters are significant differences at *p* < 0.05; the same letters indicate no differences at *p* > 0.05.

AAMs supplementation changed the amino acid content of colostrum (Table 5). AAMs1 (Gln + Leu) significantly increased the contents of Leu and Glu. AAMs3 (Leu + γ-GABA) and AAMs4 (Gln + Leu + γ-GABA) significantly increased the contents of Leu, Gly and Ala. As shown in Figure 1, all AAMs groups had significantly increased lactoprotein, total solid and IgA levels than the control group. Additionally, the concentrations of lactose and IgG in the AAMs2 (Gln + Leu), AAMs3 (Leu + γ-GABA) and AAMs4 (Gln + Leu + γ-GABA) groups were higher than these in the control group (*p* < 0.05). In terms of antioxidant indicators, Gln and Leu supplementation for the sow diets had higher activity of T-SOD and lower content of MDA than the control group (*p* < 0.05). Compared with the control group, Gln and Leu supplementation enhanced the activity of T-AOC and T-SOD (*p* < 0.05). The activity of T-AOC and GSH-PX was obviously increased in sows from the Leu and γ-GABA group (*p* < 0.05). AAMs4 (Gln + Leu + γ-GABA) supplementation had the best antioxidative effect, as evidenced by enhanced T-AOC, GSH-PX and T-SOD activity and a reduced MDA level (*p* < 0.05).

### 3.5. Effects of AAMs Supplementation on the Growth Performance of Piglets

**Figure 2:**
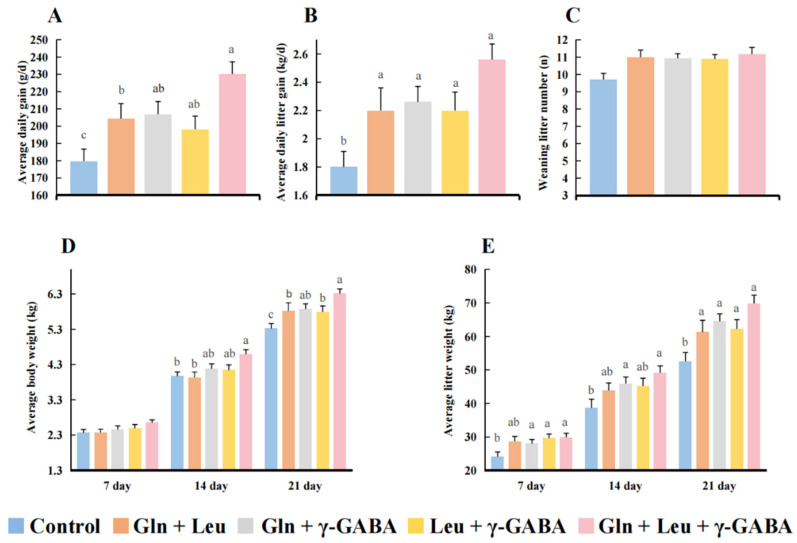
Effects of maternal AAMs supplementation during late gestation and lactation on the growth performance of their neonatal piglets (n = 10). (**A**), average daily gain; (**B**), average daily litter gain; (**C**), weaning litter number; (**D**), average body weight on days 7, 14 and 21 after birth; (**E**), average litter weight on days 7, 14 and 21 after birth. All data are presented as mean ± SEM. ^a–c^ The values with different letters are significant differences at *p* < 0.05; the same letters indicate no differences at *p* > 0.05.

Compared with the control group, piglets in all treatment groups had higher average daily gain and average daily litter gain during lactation (*p* < 0.05; Figure 2A,B). AAMs1 (Gln + Leu), AAMs2 (Gln + Leu) and AAMs3 (Leu + γ-GABA) supplementation enhanced the average litter weight of piglets on day 7 after birth (*p* < 0.05; Figure 2E). Maternal Gln and Leu (AAMs2) also increased the average litter weight of piglets on day 14 after birth (*p* < 0.05; Figure 2E). The average body weight and average litter weight of piglets on day 21 after birth in all AAMs groups were both higher than in the control group (*p* < 0.05). It is worth noting that AAMs4 supplementation showed the best growth-promoting effect.

### 3.6. Effects of AAMs Supplementation on the Serum Biochemical Parameters of Piglets

**Figure 3:**
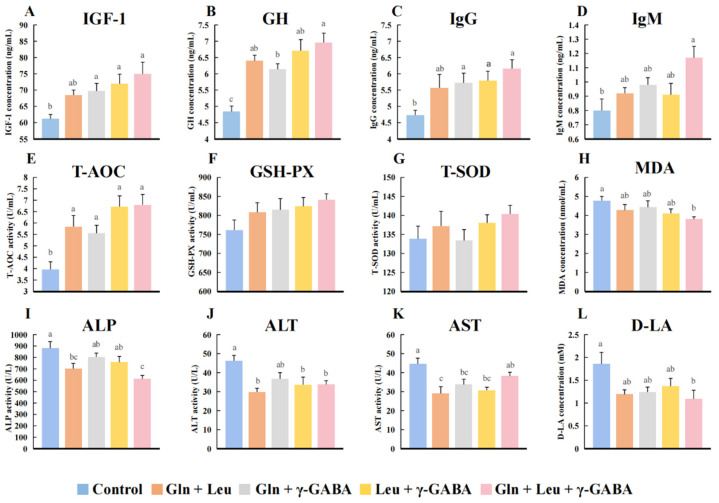
Effects of maternal AAMs supplementation during late gestation and lactation on the serum biochemical parameters of their neonatal piglets (n = 6). (**A**), IGF-1; (**B**), GH; (**C**), IgG; (**D**), IgM; (**E**), ALP; (**F**), ALT; (**G**), AST; (**H**), D-LA; (**I**), T-AOC; (**J**), GSH-PX; (**K**), T-SOD; (**L**), MDA. IGF-1, insulin-like growth factor 1; GH, growth hormone; IgG, immunoglobulin G; IgM, immunoglobulin M; ALP, alkaline phosphatase; ALT, alanine aminotransferase; AST, aspartate aminotransferase; D-LA, D-lactate; T-AOC, total antioxidant capacity; GSH-PX, glutathione peroxidase; T-SOD, total superoxide dismutase; MDA, malondialdehyde. All data were present as mean ± SEM. ^a–c^ The values with different letters are significant differences at *p* < 0.05; the same letters indicate no differences at *p* > 0.05.

The results of amino acid concentration in the serum of piglets on day 21 after birth are shown in Table 6. On day 21 after birth, serum free Gln, Leu and γ-GABA concentrations in all AAMs groups, Gly concentrations in the AAMs3 and 4 groups and His and Lys concentrations in the AAMs1 and 4 groups were higher than those in the control group (*p* < 0.05), whereas Ala concentration in the control group was higher than the AAMs1 and 4 groups (*p* < 0.05). GH and IGF-1 are important for the growth of the piglets [21,22], so we investigated the effects of AAMs supplementation on serum hormone. The concentrations of serum IGF-1 in the AAMs2, 3 and 4 groups and GH in the AAMs1, 2, 3 and 4 groups were higher than the control group (*p* < 0.05; Figure 3A,B). We also analyzed immunoglobulins concentrations and antioxidase activities to evaluate the immune and antioxidant status of piglets, respectively. On day 21 after birth, the serum IgG levels of piglets in the AAMs2, 3 and 4 groups and the serum IgM level of piglets in the AAMs4 group were higher (*p* < 0.05), compared to the control group. Compared to the control group, the serum T-AOC of piglets in the AAMs1, 2, 3 and 4 groups were higher (*p* < 0.05), while the MDA concentration of piglets in the AAMs4 group was lower (*p* < 0.05; Figure 3E–G). In addition, we evaluated the liver and intestine function of piglets by analyzing ALP, ALT, AST and D-LA. Maternal AAMs supplementation decreased the serum ALP activity of piglets in the AAMs2 and 4 group; the serum ALT activity of piglets in AAMs1, 3 and 4; and the AST activity of piglets in the AAMs1 groups (Figure 3I–K). The serum D-LA concentration of piglets in the AAMs4 group was lower (*p* < 0.05) (Figure 3L).

## 4. Discussion

The metabolic intensity of sows increases greatly during late gestation, which causes the imbalance of the redox system in sows and then affects their reproductive performance and the growth of offspring. The present study demonstrated that maternal AAMs supplementation improved the reproductive performance of sows and the growth performance of piglets. The results further showed that these beneficial effects are mediated by the improved colostrum composition and enhanced antioxidant capacity.

Sows during late gestation have intensive metabolism and mobilization, producing a large number of oxygen free radicals that destroys the balance of the body’s oxidation reduction system [4], which can be subsequently associated with the intrauterine growth restriction of the fetus [23]. SOD and GSH-PX play important roles in eliminating excess oxygen free radicals and reducing oxidative damage in animals [23]. Leu could activate the Nrf2-related signaling pathway, thus promoting the expression of antioxidant enzymes SOD2, catalase and heme oxygenase-1 [24]. Dietary Gln supplementation could improve the resistance of broiler muscle to oxidative damage through the expression of the Nrf2-Keap1 signaling pathway [25]. Maternal GABA supplementation could upregulate the GABA receptor, thus enhancing the SOD activity of sows and alleviate placental oxidative stress [26]. In the present study, higher levels of serum T-AOC, T-SOD and GSH-PX in AAMs groups on day 110 of gestation were observed, indicating the antioxidant capacity of sows was improved. Studies have shown that the enhancement of maternal antioxidant capacity is beneficial for the growth and development of offspring [8,26]. At the same time, we also observed that the serum amino acid including Leu, Glu and Lys concentrations of sows supplemented with AAMs were increased. Amino acids are crucial to the development of the fetus, and most amino acids are supplied from the maternal circulation to the fetuses by activating transport across the placenta [27]. Higher serum free AA concentrations of sows in AAMs groups on day 110 of gestation suggest increasing AA supply for fetuses. Leu has been shown to alter placental metabolism and promote maternal–fetal nutrient transport via PI3K/AKT/mTOR signaling pathway [28]. Glu provides a precursor for the synthesis of GSH-PX, which is involved in antioxidant reactions [29]. Therefore, we inferred that the improvement of antioxidant capacity and the increase of AA supply in sows during late gestation not only improved the maternal physiological status but also enhanced the litter performance to a certain extent, which was reflected in the increasing trend of healthy litter numbers in the AAMs groups.

Although the effects of AAMs supplementation on the litter performance of sows was not significant, the improvement in the growth performance of piglets in the AAMs group during lactation was noteworthy. In this study, all AAMs supplementation increased the growth performance of piglets. Among them, the combination of Gln, leu and γ-GABA had the best growth-promoting effect. Interestingly, it has been demonstrated that supplementing Gln in a rabbit diet had no positive effect on the litter weight at weaning [30]. There was no significant change in the average daily gain of weaned male pigs supplemented with γ-GABA for 35 days [31]. There was no effect on the growth performance of broilers fed with Leu [32]. It might suggest that AAMs supplementation can easier benefit piglet growth performance compared to supplementing with single AA.

Next, we explored the potential pathways of AAMs supplementation to improve growth performances in piglets. The growth of animals is largely affected by nutritional status and hormonal activity [21]. Breast milk is the main nutrient source for piglets during the neonatal period. In our study, maternal AAMs supplementation enhanced the colostrum lactoprotein, lactose and total solid contents, meaning more available nutrients for neonatal piglets. Milk protein synthesis by the mammary gland requires the absorption of large amounts of amino acids from the blood. The requirements for Leu, Arg and Lys reached more than 30 g/d [33,34]. Our results showed that all AAMs supplementation increased serum Lys levels in sows and Leu + γ-GABA supplementation increased serum Arg level. Lys is the first limiting amino acid of lactating sows [35]. Therefore, the increase in the Lys level may be an important reason for the increase of milk protein content. In addition, Arg can improve lactation performance by enhancing mammary tissue growth and nutrient uptake through the production of NO, a major vasodilator and angiogenic factor [36]. Growth and development are also regulated by GH and IGF-1. The serum levels of GH and IGF-1 were found to be positively correlated with the growth of the piglets [21,22]. In our study, higher serum GH and IGF-1 levels in AAMs groups indicated that these piglets had higher anabolic activity during lactation and can promote their growth and development.

Birth oxidative stress is an oxidative response to a sudden transition process from maternal mediated respiration in the uterus to autonomous pulmonary respiration outside the uterus. It is confirmed that piglets suffer from heavy oxidative stress after birth, which causes oxidative damage to lipids, proteins and DNA [37]. AAMs supplementation can increase the level of T-AOC in the colostrum and serum of piglets, which is helpful for neonatal piglets to resist the adverse effects of oxidative damage. In addition, after the piglets are born, microbes begin to colonize the intestine. The incomplete development of intestinal function in the newborn stage is prone to the invasion of pathogenic microorganisms, which can cause intestinal barrier and liver damage [38,39]. Therefore, neonatal piglets must acquire maternal immunoglobulins from the colostrum for passive immune protection [40]. Gln is an important energy and biosynthetic nutrient for the proliferation, survival and function of B cells, which are important immunoglobulin producers [41,42]. A study has reported that GABA supplementation can also increase serum immunoglobulin content in hens. The probable reason is the inhibition of GABA on somatostatin and adrenal corticosteroid hormone secretion [43], which impairs immunoglobulin. Therefore, dietary AAMs supplementation could enhance the IgA and IgG levels in colostrum, thereby helping piglets obtain passive immunity. IgA plays an important role in regulating intestinal microbiota and preventing early intestinal inflammation, which could distribute in mucosa to prevent the invasion of pathogenic microorganisms after entering the intestine [44]. IgG largely recognizes virulence factors encoded within the locus of the enterocyte effacement pathogenicity island, including the adhesin Intimin and T3SS filament EspA, which are major antigens conferring protection [45]. Thus, IgG in breast milk protects neonates against infection with an attaching and effacing pathogen. These may also explain that AAMs supplementation could reduce serum ALP, ALT, AST and D-LA levels. The results showed that AAMs supplementation increased immunoglobulin content in colostrum, thereby improving the intestinal barrier function and liver function of piglets.

## 5. Conclusions

This study focused on the issue of oxidative stress suffered by sows during late gestation and lactation and evaluated the effects of supplementation with an amino acid mixture on the oxidative stress and growth performance of the offspring. In our study, maternal supplementation with the combined Gln, Leu and γ-GABA during late gestation and lactation enhanced the antioxidant capacity and colostrum composition of the sows, thus improving the antioxidant capacity and growth performances of neonatal piglets. Furthermore, maternal supplementation with AAMs4 (0.55% Gln + 0.44% Leu + 0.01% γ-GABA) showed the best effect. The study provides new insight into a nutritional strategy that can alleviate oxidative stress in sows and improve the growth performance of newborn piglets. Although this study demonstrated that AAMs supplementation can improve the performance of sows and their piglets, the specific mechanism of action remains to be further clarified.

## Figures and Tables

**Table 1:** Basal diet composition and nutrition level of gestational and lactating sows.

Items	Gestation	Lactation
Ingredient amount (%)		
Corn	64.84	64.97
Soybean meal	16.70	16.00
Wheat bran	10.00	10.50
Soybean oil	4.00	4.00
Salt	0.30	0.30
Premix ^1^	4.00	4.00
L-lysine (98 %)	0.10	0.15
L-threonine (98 %)	0.04	0.05
L-tryptophane (98 %)	0.02	0.03
Total	100	100
Nutrition level (calculated value ^2^)		
Digestible energy (Mcal/kg)	3.38	3.40
Crude protein (%)	14.41	15.21
Calcium (%)	0.83	0.72
Total phosphorus (%)	0.64	0.62
L-lysine (%)	0.75	0.84
L- methionine (%)	0.22	0.23
L- methionine+ L-cysteine, %	0.47	0.49
L-threonine (%)	0.52	0.56
L-tryptophane (%)	0.16	0.18

^1^ Composition of 1 kg premix: 240–300 KIU VA, 50–125 KIU VD3, 500 IU VE, ≥45 mg VK3, ≥50 mg VB1, ≥150 mg VB2, ≥100 mg VB6, ≥0.5 mg VB12, ≥650 mg niacin, ≥450 mg pantothenic acid, ≥80 mg folic acid, ≥10 mg biotin, 2.4–18 g iron, 0.2–0.62 g copper, 1–2.5 g zinc, 0.5–2 g manganese, 10–50 mg iodine, 5–12.5 mg selenium, 60–220 g sodium chloride, ≥10 mg choline chloride, ≥0.7% L-lysine, ≤10% water. ^2^ Calculated value: the nutrition levels of basal diet were calculated by an analysis of the crude protein, calcium, total phosphorus and various digestible amino acid levels of corn, soybean meal and wheat bran and DE recommended by NRC.

**Table 2:** Effects of maternal AAMs supplementation during late gestation and lactation on the litter performance and feed intake of the sows.

Items	Treatments					*p*-Value
	Control	Gln + Leu	Gln + γ-GABA	Leu + γ-GABA	Gln + Leu + γ-GABA	
Average of parity	1.08 ± 0.08	1.00 ± 0.00	1.14 ± 0.10	1.18 ± 0.18	1.17 ± 0.08	0.788
Number of piglets born	11.31 ± 0.92	12.91 ± 0.67	11.78 ± 0.69	11.72 ± 0.67	12.50 ± 0.52	0.546
Number of piglets born alive	11.15 ± 0.90	12.82 ± 0.60	11.71 ± 0.71	11.36 ± 0.63	12.00 ± 0.51	0.530
Number of healthy piglets	8.54 ± 0.83	10.55 ± 0.70	10.64 ± 0.67	10.72 ± 0.37	10.83 ± 0.76	0.053
Birth weight (kg)	1.33 ± 0.09	1.30 ± 0.05	1.40 ± 0.06	1.42 ± 0.05	1.35 ± 0.05	0.723
Birth litter weight (kg)	14.44 ± 1.04	16.73 ± 1.03	16.48 ± 0.77	15.97 ± 1.06	16.14 ± 0.98	0.507
Lactation ADFI (kg/d)	4.51 ± 0.14 ^b^	5.06 ± 0.18 ^ab^	4.77 ± 0.21 ^b^	4.97 ± 0.29 ^b^	5.67 ± 0.25 ^a^	0.007

Results are expressed as means ± SEM. ^a,b^ The values with different letters are significant differences at *p* < 0.05; the same letters indicate no differences at *p* > 0.05. The number of repetitions for all 5 treatment groups was 10. ADFI, average daily feed intake. Healthy piglet: birth weight ≥ 1 kg.

**Table 3:** Effects of maternal AAMs supplementation during late gestation and lactation on serum free amino acids of sows on G110.

Items (µmol/L)	Treatments					*p*-Value
	Control	Gln + Leu	Gln + γ-GABA	Leu + γ-GABA	Gln + Leu + γ-GABA	
Glutamine	1180.54 ± 48.83	1306.69 ± 39.02	1367.57 ± 132.74	1221.93 ± 91.70	1552.03 ± 100.39	0.056
Leucine	183.64 ± 6.87 ^c^	229.58 ± 12.81 ^b^	196.47 ± 4.03 ^c^	268.86 ± 13.42 ^a^	247.55 ± 12.40 ^ab^	<0.01
γ-aminobutyric acid	96.51 ± 4.82	103.87 ± 3.15	116.19 ± 5.09	106.25 ± 5.25	106.42 ± 2.13	0.052
Glycine	483.98 ± 38.61	484.95 ± 62.64	619.09 ± 72.24	464.77 ± 44.28	539.58 ± 56.71	0.317
Alanine	504.95 ± 36.07	405.32 ± 45.98	395.16 ± 56.36	414.85 ± 33.42	494.37 ± 53.19	0.301
Serine	116.42 ± 16.00	110.08 ± 13.13	118.90 ± 18.20	102.48 ± 12.74	88.19 ± 5.83	0.535
Proline	266.90 ± 45.05	276.97 ± 28.45	310.04 ± 52.28	387.01 ± 56.41	328.98 ± 30.56	0.343
Valine	260.53 ± 28.92	175.61 ± 12.68	219.13 ± 38.62	200.31 ± 22.45	191.18 ± 21.10	0.218
Threonine	1458.00 ± 49.69	1260.26 ± 43.36	1368.32 ± 106.02	1474.89 ± 129.42	1368.18 ± 66.62	0.425
Glutamic acid	120.58 ± 5.41 ^c^	139.26 ± 8.55 ^bc^	124.71 ± 7.61 ^c^	155.01 ± 12.36 ^b^	178.89 ± 11.19 ^ab^	0.001
Methionine	40.41 ± 1.24	45.73 ± 2.41	46.54 ± 1.51	43.94 ± 1.84	50.49 ± 3.49	0.055
Histidine	16.90 ± 0.17	15.82 ± 0.52	17.43 ± 0.90	16.44 ± 0.90	16.69 ± 0.61	0.562
Phenylalanine	66.91 ± 4.20 ^b^	70.92 ± 7.72 ^b^	75.34 ± 4.16 ^b^	72.65 ± 3.00 ^b^	90.21 ± 3.39 ^a^	0.022
Arginine	186.00 ± 7.28 ^b^	195.83 ± 9.83 ^b^	201.90 ± 7.39 ^b^	250.34 ± 9.78 ^a^	193.58 ± 6.30 ^b^	<0.01
Tyrosine	122.11 ± 5.87	112.30 ± 6.08	120.64 ± 9.93	118.85 ± 8.78	101.17 ± 4.73	0.314
Lysine	65.45 ± 2.20 ^d^	78.98 ± 2.75 ^c^	80.72 ± 4.79 ^c^	108.73 ± 2.42 ^a^	92.40 ± 2.19 ^b^	<0.01

Results are expressed as means ± SEM. ^a–d^ The values with different letters are significant differences at *p* < 0.05; the same letters indicate no differences at *p* > 0.05. The number of repetitions for all 5 treatment groups was 6.

**Table 4:** Effects of maternal AAMs supplementation during late gestation and lactation on the antioxidant capacity of sows on G110.

Items	Treatments					*p*-Value
	Control	Gln + Leu	Gln + γ-GABA	Leu + γ-GABA	Gln + Leu + γ-GABA	
T-AOC (U/mL)	5.90 ± 0.43 ^b^	6.99 ± 0.45 ^ab^	7.23 ± 0.44 ^a^	7.44 ± 0.20 ^a^	8.06 ± 0.37 ^a^	0.011
GSH-PX (U/mL)	684.40 ± 47.34 ^b^	788.99 ± 48.19 ^ab^	908.25 ± 25.06 ^a^	781.65 ± 49.43 ^ab^	873.39 ± 42.03 ^a^	0.011
T-SOD (U/mL)	145.95 ± 3.12 ^b^	156.36 ± 4.10 ^ab^	146.63 ± 1.72 ^b^	159.85 ± 2.57 ^a^	155.67 ± 4.46 ^ab^	0.022
MDA (nmol/mL)	4.28 ± 0.28 ^a^	2.92 ± 0.43 ^bc^	3.64 ± 0.21 ^ab^	2.43 ± 0.28 ^c^	2.56 ± 0.38 ^bc^	0.002

Results are expressed as means ± SEM. ^a–c^ The values with different letters are significant differences at *p* < 0.05; the same letters indicate no differences at *p* > 0.05. T-AOC, total antioxidant capacity; GSH-PX, glutathione peroxidase; T-SOD, total superoxide dismutase; MDA, malondialdehyde. The number of repetitions for all 5 treatment groups was 6.

**Table 5:** Effect of maternal AAMs supplementation during late gestation and lactation on the free amino acids of the colostrum.

Items (µmol/L)	Treatments					*p*-Value
	Control	Gln + Leu	Gln + γ-GABA	Leu + γ-GABA	Gln + Leu + γ-GABA	
Glutamine	137.93 ± 17.48	203.66 ± 20.51	159.60 ± 12.16	154.58 ± 13.66	167.54 ± 13.27	0.064
Leucine	28.92 ± 2.0 ^c^	44.96 ± 5.49 ^ab^	37.57 ± 3.33 ^bc^	36.34 ± 2.55 ^b^	54.31 ± 4.63 ^a^	<0.01
γ-aminobutyric acid	68.08 ± 1.44	74.55 ± 2.87	71.36 ± 0.66	71.04 ± 0.39	70.67 ± 0.30	0.062
Glycine	20.55 ± 1.19 ^c^	21.50 ± 1.44 ^c^	22.55 ± 1.69 ^bc^	32.02 ± 1.93 ^a^	27.06 ± 2.24 ^ab^	<0.01
Alanine	43.47 ± 2.87 ^a^	37.60 ± 2.33 ^ab^	36.76 ± 3.20 ^ab^	33.00 ± 3.08 ^bc^	24.92 ± 2.77 ^c^	0.001
Serine	32.96 ± 1.65 ^b^	34.33 ± 2.76 ^ab^	36.40 ± 2.16 ^a^	31.51 ± 1.96 ^ab^	37.91 ± 3.04 ^a^	0.334
Proline	64.07 ± 3.52	76.60 ± 5.84	68.13 ± 4.68	64.22 ± 5.18	73.27 ± 5.51	0.312
Valine	34.70 ± 4.00 ^c^	36.78 ± 3.25 ^ab^	33.94 ± 4.46	30.60 ± 2.23 ^bc^	40.72 ± 3.79 ^a^	0.387
Glutamic acid	42.09 ± 3.29 ^b^	65.61 ± 5.05 ^a^	51.42 ± 3.97 ^b^	50.82 ± 3.29 ^b^	53.33 ± 5.96 ^ab^	0.015
Methionine	7.25 ± 0.40	6.93 ± 0.34	6.59 ± 0.25	6.42 ± 0.24	6.10 ± 0.37	0.141
Histidine	11.33 ± 0.46	11.50 ± 0.36	10.47 ± 0.13	10.63 ± 0.07	10.96 ± 0.25	0.079
Phenylalanine	26.23 ± 1.99	24.28 ± 2.07	21.83 ± 0.95	23.26 ± 1.55	20.62 ± 1.13	0.139
Arginine	6.28 ± 0.20	6.09 ± 0.21	6.11 ± 0.04	6.19 ± 0.09	6.17 ± 0.08	0.873
Tyrosine	27.83 ± 2.07	25.15 ± 2.41	23.89 ± 1.76	23.33 ± 1.26	24.80 ± 1.83	0.509
Lysine	20.99 ± 1.74	19.21 ± 0.46	18.37 ± 0.75	17.07 ± 0.60	18.49 ± 1.59	0.208

Results are expressed as means ± SEM. ^a–c^ The values with different letters are significant differences at *p* < 0.05; the same letters indicate no differences at *p* > 0.05. The number of repetitions for all 5 treatment groups was 10.

**Table 6:** Effects of maternal AAMs supplementation during late gestation and lactation on the free amino acids of neonatal piglets on day 21 after birth.

Item (µmol/L)	Treatments					*p*-Value
	Control	Gln + Leu	Gln + γ-GABA	Leu + γ-GABA	Gln + Leu + γ-GABA	
Glutamine	1061.70 ± 56.65 ^b^	1897.68 ± 116.92 ^a^	1658.13 ± 228.91 ^a^	1662.83 ± 130.77 ^a^	1890.09 ± 119.61 ^a^	0.002
Leucine	143.62 ± 10.74 ^b^	188.95 ± 8.84 ^a^	172.77 ± 9.30 ^a^	171.01 ± 5.65 ^a^	173.29 ± 9.14 ^a^	0.024
γ-aminobutyric acid	61.72 ± 0.011 ^c^	84.81 ± 2.63 ^b^	97.48 ± 4.60 ^a^	91.43 ± 3.58 ^ab^	90.28 ± 2.39 ^ab^	<0.01
Glycine	529.21 ± 27.89 ^c^	684.80 ± 58.56 ^bc^	563.33 ± 23.77 ^bc^	709.30 ± 71.29 ^ab^	776.79 ± 47.67 ^a^	0.013
Alanine	717.70 ± 68.70 ^a^	455.75 ± 18.05 ^b^	660.89 ± 32.21 ^a^	673.01 ± 15.72 ^a^	523.80 ± 28.43 ^b^	<0.01
Serine	124.89 ± 6.36	169.56 ± 13.6	136.91 ± 11.01	149.22 ± 12.67	148.22 ± 19.28	0.204
Proline	339.15 ± 28.01	472.62 ± 16.01	353.71 ± 25.90	496.89 ± 99.89	474.75 ± 37.86	0.113
Valine	250.42 ± 13.64	251.96 ± 7.39	225.79 ± 17.53	223.59 ± 29.83	211.25 ± 19.03	0.487
Threonine	448.03 ± 39.17	502.12 ± 36.26	448.55 ± 19.07	572.15 ± 55.78	470.43 ± 30.02	0.149
Glutamic acid	150.77 ± 26.16	178.70 ± 7.71	118.13 ± 15.01	143.29 ± 15.51	153.07 ± 31.23	0.388
Methionine	36.30 ± 3.60	52.34 ± 3.94	53.10 ± 5.73	48.28 ± 6.34	56.13 ± 4.58	0.073
Histidine	59.65 ± 5.09 ^ab^	72.39 ± 3.79 ^a^	61.78 ± 4.24 ^ab^	54.15 ± 5.39 ^b^	72.75 ± 4.08 ^a^	0.029
Phenylalanine	83.46 ± 5.20	100.57 ± 3.21	99.71 ± 9.21	95.20 ± 4.87	102.54 ± 7.67	0.252
Arginine	8.60 ± 0.27	9.83 ± 0.33	9.91 ± 0.55	9.94 ± 0.46	9.85 ± 0.58	0.214
Tyrosine	108.91 ± 10.48	147.46 ± 9.52	124.96 ± 15.55	122.46 ± 13.62	156.42 ± 21.17	0.170
Lysine	116.31 ± 11.67 ^b^	169.32 ± 12.33 ^a^	152.80 ± 15.03 ^ab^	132.96 ± 12.67 ^ab^	163.12 ± 7.25 ^a^	0.026

Results are expressed as means ± SEM. ^a–c^ The values with different letters are significant differences at *p* < 0.05; the same letters indicate no differences at *p* > 0.05. The number of repetitions for all 5 treatment groups was 6.

## Data Availability

The data presented in this study are available in the article.
